# Longitudinal CNS and systemic T-lymphocyte and monocyte activation before and after antiretroviral therapy beginning in primary HIV infection

**DOI:** 10.3389/fimmu.2025.1531828

**Published:** 2025-02-25

**Authors:** Phillip Chan, Xiang Li, Fangyong Li, Brinda Emu, Richard W. Price, Serena Spudich

**Affiliations:** ^1^ Department of Neurology, Yale University School of Medicine, New Haven, CT, United States; ^2^ Yale Center for Brain and Mind Health, Yale University School of Medicine, New Haven, CT, United States; ^3^ Yale Center for Analytical Sciences, Yale University School of Medicine, New Haven, CT, United States; ^4^ Department of Medicine, Division of Infectious Diseases, Yale School of Medicine, New Haven, CT, United States; ^5^ Department of Neurology, University of California, San Francisco, San Francisco, CA, United States

**Keywords:** primary HIV infection, antiretroviral therapy, cerebrospinal fluid, T-lymphocyte, monocyte

## Abstract

**Background:**

Trafficking of immune cells to the central nervous system is hypothesized to facilitate HIV entry and immune-induced neuronal injury and is mediated by surface proteins such as chemokine receptors and α4 integrin. We longitudinally assessed immune cell activation and surface marker expression in cerebrospinal fluid (CSF) and blood and their relationship with CSF HIV RNA beginning during primary HIV infection (PHI) before and after antiretroviral therapy (ART).

**Methods:**

Longitudinal paired blood and CSF were obtained in ART-naïve PHI (<12 month since infection) participants; some independently initiated ART during follow up. Multiparameter flow cytometry of fresh samples determined activation (% CD38^+^HLADR^+^) and chemokine receptor expression (% CCR5^+^ and CXCR3^+^) on CD4^+^ and CD8^+^ T cells, and subtype and α4 integrin expression (% and mean fluorescence intensity (mfi) of CD49d^+^) on monocytes. HIV RNA was quantified by PCR. Analyses employed Spearman correlation, within-subject correlation, and linear mixed models.

**Results:**

51 participants enrolled at a median 3.2 months post HIV transmission with 168 total visits (113 pre-ART, 55 post-ART) and a median of 6.5 months of longitudinal follow up (range 0-40). In pre-ART PHI, frequencies of activated CD4+ and CD8+ T cells were much higher in CSF than in blood, with levels similar to ART-naïve people with chronic HIV infection. Both CSF CD4+ and CD8+ T cell activation increased longitudinally prior to initiation of ART. In multivariate analysis, CSF CD4+ but not CD8+ T cell activation independently predicted CSF HIV RNA. Neither CSF monocyte subtypes or α4 expression correlated with CSF HIV RNA. Blood monocyte α4 MFI correlated with CD4+ and CD8+ T cell activation (p<0.05). Following ART initiation, blood but not CSF T cell activation declined with days on treatment (slope=-0.06, p=0.001). During ART, blood and CSF monocyte α4 MFI correlated with T cell activation (p<0.05).

**Conclusions:**

In untreated early infection after PHI, immune activation increases over time, and CSF CD4+ T cell activation but not monocyte activation correlates with CSF HIV RNA. Intrathecal T cell activation does not decline during early follow up on ART. Immunomodulating therapies may be needed to prevent neuronal injury and HIV neuroinvasion during early HIV.

## Introduction

HIV is a multi-organ disease that involves the central nervous system (CNS) (1). Prior investigation confirms that HIV invades the CNS within days after transmission (2–4) and establishes infection of cells residing in the CNS within the first year after acquisition and that evolves throughout untreated infection (5–7). This local infection of CNS cells persists despite antiretroviral therapy (ART) (8–12). Both systemic and intrathecal immunologic responses are readily observed during Fiebig I-V acute HIV infection (AHI) (13), including immune cell activation and elevation of immune activation markers in both blood and cerebrospinal fluid (CSF) compartments (4, 14). While these findings demonstrate the onset of HIV neuropathogenesis during AHI, studies during primary HIV infection (PHI), defined as within the first year of infection following acquisition, highlight that several adverse CNS events may happen after AHI and during the early phases of infection. Sequencing studies have revealed the development of compartmentalized quasispecies in the CSF during PHI, but not AHI (5, 15–17), suggesting the establishment of local viral replication within the CNS during this period. Abnormal levels of CSF neurofilament light chain (NFL), a protein biomarker reflective of active injury to neurons, has been detected in up to 40% of CSF samples collected from untreated people with HIV (PWH) during PHI, in association with neuroimaging evidence of reduced neuronal integrity (18). Moreover, soluble markers of immune activation and blood brain barrier compromise are both elevated during early infection and increase in the first few years following initial HIV infection in the absence of treatment with ART (19, 20). Diffusion tensor imaging (DTI) further reveals that white matter and grey matter structural changes manifest in the brain during PHI (21, 22) and that regional volumes in the brain decrease over time with duration of infection after PHI (23). These findings suggest that PHI is a highly evolutionary period of HIV neuropathogenesis, during which timely ART initiation may alter the trajectory of neurological outcomes.

It remains unclear how immune cell states in the blood and CSF compartments evolve and contribute to CNS HIV infection during PHI prior to and after ART. These include cells that are known to be pathogenically important in HIV, such as CD4+ T-cells, monocytes, and cytotoxic CD8+ T-cells. The former two are susceptible to HIV infection and contribute to HIV replication, while the latter are responsible for limiting HIV replication and at the same time associating with adverse inflammation. Recent studies have suggested that CD4+ T cells may be target cells of infection not only in the periphery but also in the CNS during acute HIV, and may importantly contribute to establishment and maintenance of CNS HIV reservoirs (24–27). To date, evaluating the natural course of immunological changes during PHI and early HIV infection has posed significant challenges. First, identifying HIV infection within the first year after transmission is difficult, as PWH are mostly asymptomatic during the period. Second, as immediate ART upon HIV diagnosis provides the best outcomes for PWH (28), observational studies deferring ART initiation are no longer feasible.

Leveraging a prospective study of paired blood and CSF samples collected before guidelines for implementation of immediate ART, this analysis aimed to examine longitudinal changes in CD4+ and CD8+ T-cell and monocyte phenotypes in the respective compartments beginning during PHI both prior to and following ART initiation. Comparison participants included cross-sectionally collected paired samples from pre-ART PWH with chronic HIV infection (CHI, defined as known HIV diagnosis for at least three years) and people without HIV (PWoH). Laboratory evaluations on paired samples included measurements of activated CD4+ and CD8+ T-cells frequency, monocyte subpopulation composition, and alpha-4 (α4) integrin expression in monocytes, which signifies monocytes’ potential trafficking capacity across compartments via cell adhesion and transmigration. Finally, potential correlations between T-cell activation and monocyte subpopulations, both before and after ART initiation, were explored.

## Materials and methods

### Study design and participants

PHI study participants were enrolled in the Primary Infection Stage CNS Events Study (PISCES) cohort study conducted in San Francisco between 2005 and 2014, including 51 pre-ART PHI participants and 11 age and gender matched PWoH. In addition, data collected using identical methods from 32 initially pre-ART CHI participants enrolled in a separate observational study in San Francisco was included for comparison. In most participants, PHI was confirmed by documentation of a negative HIV test within the previous 12 months followed by a positive test, where estimated date of infection was designated 14 days prior to the onset of an acute retroviral syndrome, or in asymptomatic participants as the halfway point between the last negative and first positive test. In a minority of participants, recent infection was confirmed by a participant reporting a recent exposure, an acute retroviral like syndrome, and evidence of infection within the past six months by a diluted (less sensitive) HIV antibody test, as described in previous reports (20, 29). All PHI participants were ART-naïve at baseline and were followed at 6 weeks, and then every 6 months thereafter. As PISCES was established before guidelines recommended immediate ART initiation for all PWH regardless of CD4+ T-cell levels, the timing of ART initiation among PHI participants varied individually, depending on decisions made by the participants and their clinicians. The settings resulted in the collection of serial CSF samples from PHI participants both before and after ART. Compared to longitudinal CSF samplings among participants with PHI, CHI and PWoH participants served as study controls and underwent cross-sectional CSF sampling (29). Informed consent was obtained from all participants. The PISCES study protocol was approved by the University of California San Francisco Committee on Human Research (H9133-26278).

### Sample collection & laboratory procedures

Paired blood and CSF samples were obtained from all study participants at baseline, and from PHI participants during follow-up visits. Blood CD4+ and CD8+ T-cell count, CSF white blood cell (WBC) count, protein and albumin were measured using fresh samples. Blood and CSF HIV-1 RNA were measured in previously frozen (-70°C) cell free samples, using the ultrasensitive Amplicor HIV Monitor (version 1.5; Roche Molecular Diagnostic Systems, Branchburg, NJ) assay (29).

### Flow cytometry

Paired blood and CSF samples were prepared as previously described (30, 31). Briefly, multiparameter flow cytometry was performed on fresh samples of whole blood and the cellular component of CSF (separated by centrifugation from the cell free fraction) to assess the percentage of activated CD4^+^ and CD8^+^ T-cells in the samples, based on CD38 and HLA-DR co-expression (32). Blood and CSF monocytes were classified by CD14 and CD16 expression, while expression of α4 integrin was measured as the percentage of CD49d^+^ monocytes and the mean/median fluorescence intensity (MFI) of CD49d staining.

Since low monocyte percentages in CSF precluded splitting CSF cell pellet samples, we chose to examine a subset of samples for T-cell flow cytometry markers including analysis of CCR5+ and CXCR3+ expression in T-cells (Panel 1), and in 2008, flow cytometry antibody-dye panels were switched in order to study monocyte markers including expression of α4 integrin in monocytes (Panel 2). Participants already enrolled by 2008 continued to have longitudinal samples analyzed using Panel 1 while newly enrolling participants in 2008 and thereafter had samples analyzed using Panel 2 (**Supplementary Tables S1**, **S2**). Comparison of the 2 panels showed differences in CSF CD4+ T-cell activation and CSF monocyte activation, hence data from the two panels were analyzed separately throughout this study. Panel 1 data was used for baseline and longitudinal analyses of T-cells. Panel 2 data was used for analyses of monocytes and for comparison of monocyte alpha-4 integrin expression with T-cell activation.

Monoclonal antibodies included CD3, CD4, CD8, CD38, HLA-DR, CCR5, CXCR3, CD45, CD14, CD16, and CD49d conjugated to allophycocyanin (APC), phycoerythrin (PE) peridinin chlorophyll protein (PerCP), fluorescein isothiocyanate (FITC), and tandem conjugations with cyanine (ACP-Cy7, PE-Cy7) and Texas Red (PE-Texas Red). Blood samples were stained with fluorescence-minus-one controls in which one antibody was omitted; an unstained control and single-stained samples were also prepared as compensation controls. Samples were run on a FACS DIVA (BD Biosciences, San Jose, CA) and flow cytometry data analyzed with FlowJo (TreeStar, Ashland, OR).

### Statistical analyses

Participant characteristics were summarized as frequency and percentage, or median and interquartile range (IQR) as appropriate. Chi-square test for categorical variables or Kruskal-Wallis test with *post hoc* testing using Dunn’s multiple comparisons for continuous variables was performed to compare groups. Longitudinal data was analyzed using linear mixed models, which accounts for correlations among repeated assessments within same individual using the effect of random intercept. Further, Sobel’s test was used for mediation analysis, to examine whether the increase in CSF CD8+ cells from the increase of CSF HIV RNA level was medicated by the increase of CSF CD4+ cells. Statistics were performed and graphics generated using IBM SPSS Statistics 25 (IBM, Armonk, NY) or Prism 7 (GraphPad Software Inc, La Jolla, CA). Statistical significance was p<0.05, two-sided.

## Results

### Participant characteristics

All 51 PHI participants were male, with a median age of 36 (IQR 31-45) years at study baseline (i.e., 1st CSF sampling, pre-ART). Please see **Table 1** for demographic information. The estimated duration of HIV infection was 3.2 (IQR 2.4-5.6) months. At baseline, plasma and CSF HIV RNA were 4.37 (IQR 3.80-4.86) and 2.31 (IQR 1.69-3.10) log_10_cps/ml, while blood CD4+ and CD8+ T-cell levels were 581 (IQR 429-738) and 985 (IQR 691-1336) cells/µl.

**Table 1:** Characteristics of primary infection participants.

	Primary HIV (n=51)
Age (years)	36 (31, 45)
Male, n (%)	51 (100)
Baseline Visit	
Estimated time post HIV transmission (months)	3.2 (2.4, 5.6)
CD4+ count (cells/µl)	581 (429, 738)
CD8+ count (cells/µl)	985 (691, 1336)
Plasma HIV RNA (log_10_ copies/ml)	4.37 (3.80, 4.86)
CSF HIV RNA (log_10_ copies/ml)	2.31 (1.69, 3.10)
CSF WBC count (cells/mm^3^)	6 (2, 11)
CSF protein (mg/dL)	41.0 (35.5, 51.3)
Longitudinal Analysis	
Follow-up duration (months)	6.5 (0, 22.7)
Total number of study visits	168
Sample included in longitudinal Analysis	
Without ART	113
With ART initiation	55
Estimated duration of HIV infection at ART initiation (months)	6.2 (2.1, 16.0)

Median (interquartile range) shown unless otherwise indicated.

PHI participants were longitudinally followed for a median duration of 6.5 (IQR 0-22.7) months. Including the baseline visit, they contributed to 168 visits with paired blood and CSF sampling. Two longitudinal analyses were constructed according to their ART status. The first one examined the immunological changes based on samples collected from PHI participants without ART initiation. The analysis included 113 paired samples, including 21 PHI participants who underwent repeated CSF sampling. The second longitudinal analysis examined the immunological changes after ART based on 55 paired samples. In the analysis, all baseline samples were collected pre-ART, whereas all subsequent samples were collected post-ART, contributed by 11 PHI participants. Of note, all post-ART samples donors achieved and maintained plasma HIV suppression during longitudinal sample collection.

### T-cell activation in blood and CSF samples at baseline

**Figure 1:**
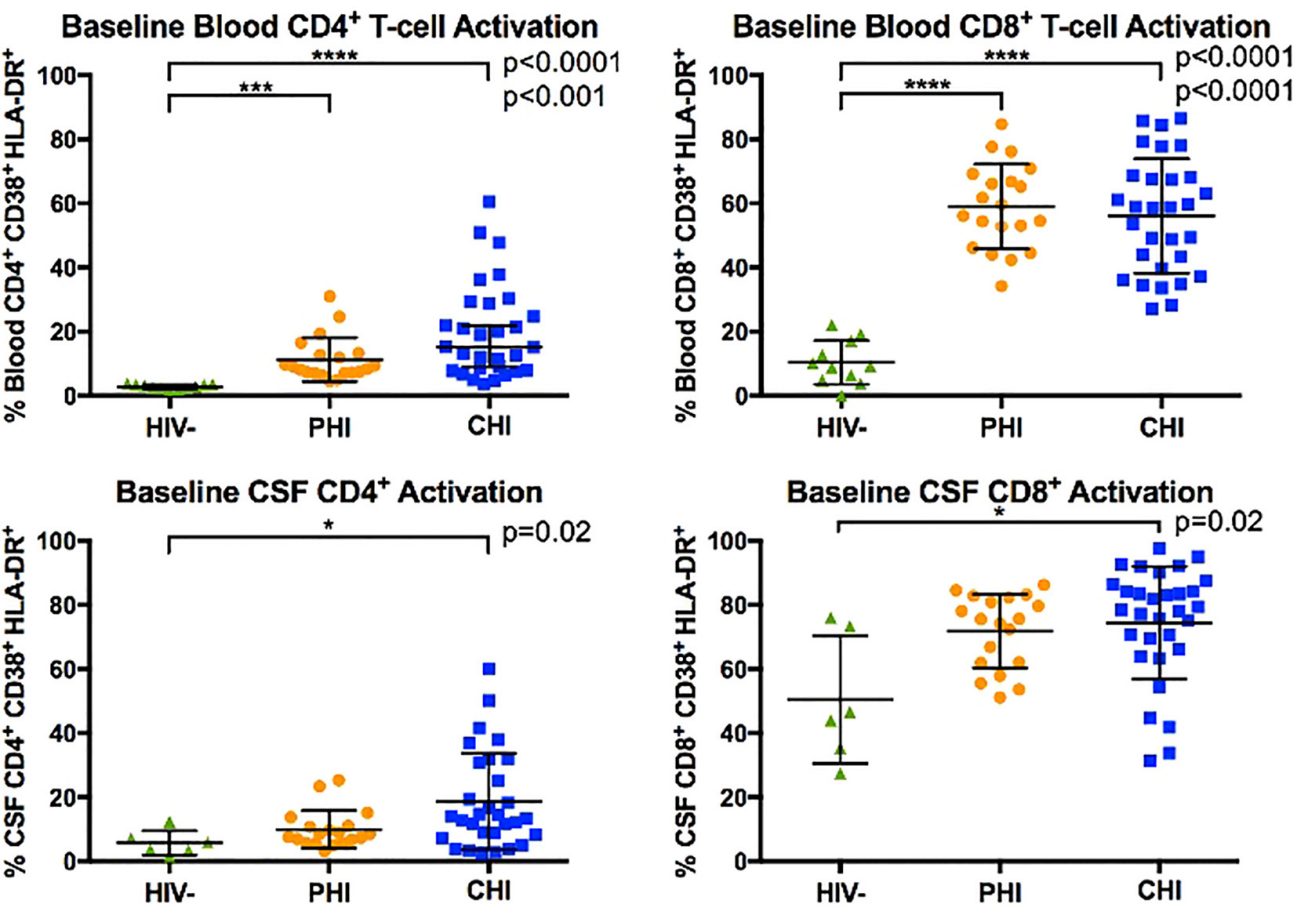
Comparison of blood and CSF CD4^+^ and CD8^+^ T-cell activation (% CD38^+^ HLA-DR^+^) between HIV-negative, PHI, and CHI participants at baseline visit. Horizontal line indicates the group mean and error bars indicate standard deviation. * p< 0.05, *** p< 0.001, **** p< 0.0001.

**Figure 1** compares the percentages of activated CD4^+^ and CD8^+^ T-cells in blood and CSF samples across the three participant groups: pre-ART PHI at baseline, pre-ART CHI and PWoH. In blood, both PHI and CHI participants exhibited higher percentages of activated CD4+ and CD8+ T-cell than PWoH (p<0.001). Percentages of activated CD4+ and CD8+ T-cells in blood did not differ statistically between PHI and CHI participants.

In CSF, CHI but not PHI participants demonstrated greater percentages of activated CD4+ and CD8+ T-cell than PWoH (p=0.02). However, percentages of activated CD4+ and CD8+ T-cells in CSF did not differ statistically between PHI and CHI participants. We interpret these data as suggesting that PHI participants had levels of T-cells activation intermediate between those of CHI and PWoH.

### Monocyte subpopulation and α4 integrin expression in blood and CSF samples at baseline

**Figure 2:**
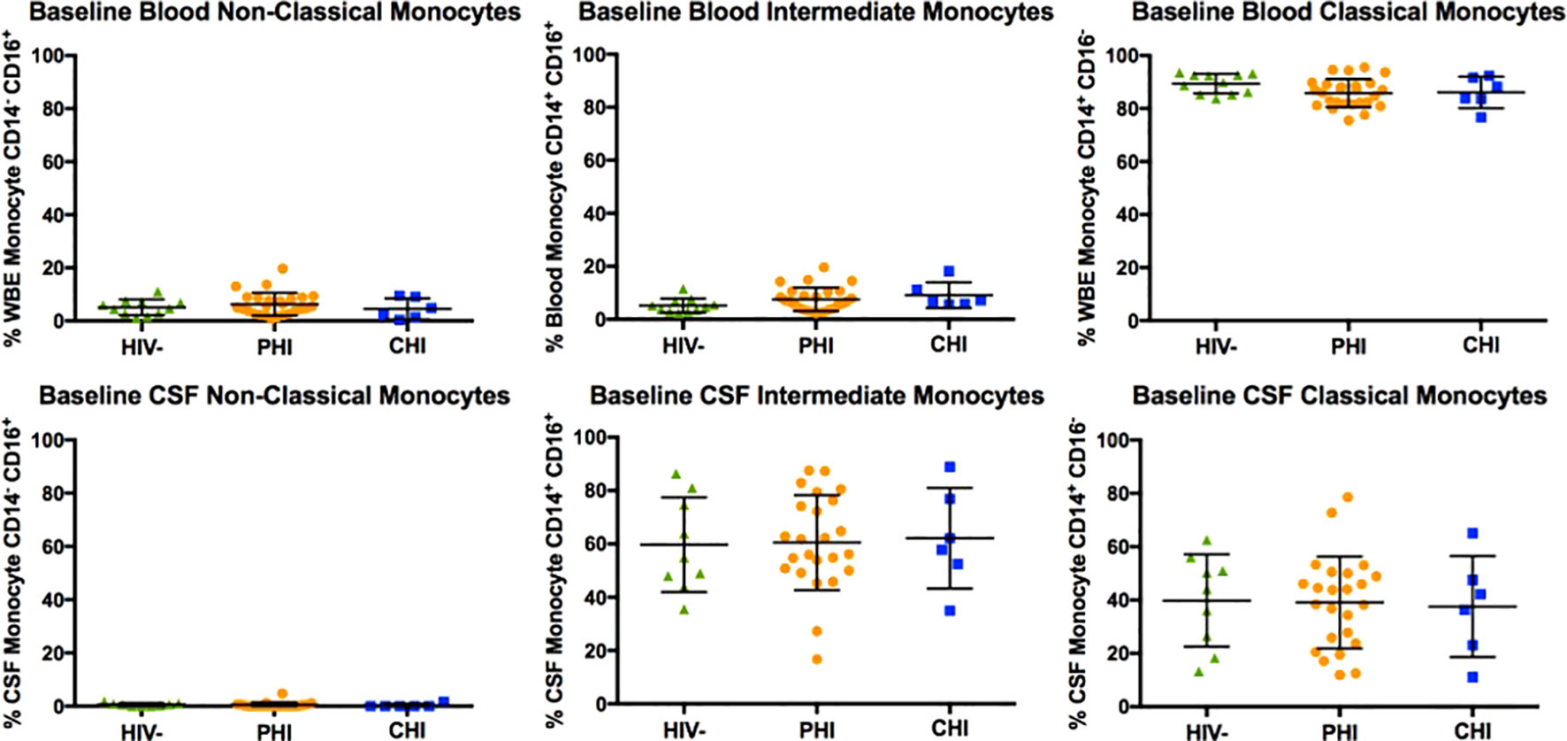
Baseline comparison of non-classical (CD14^dim^ CD16^+^), intermediate (CD14^+^ CD16^+^), and classical (CD14^+^ CD16^-^) monocyte subtypes in blood and CSF of HIV-negative, PHI, and CHI participants.

**Figure 3:**
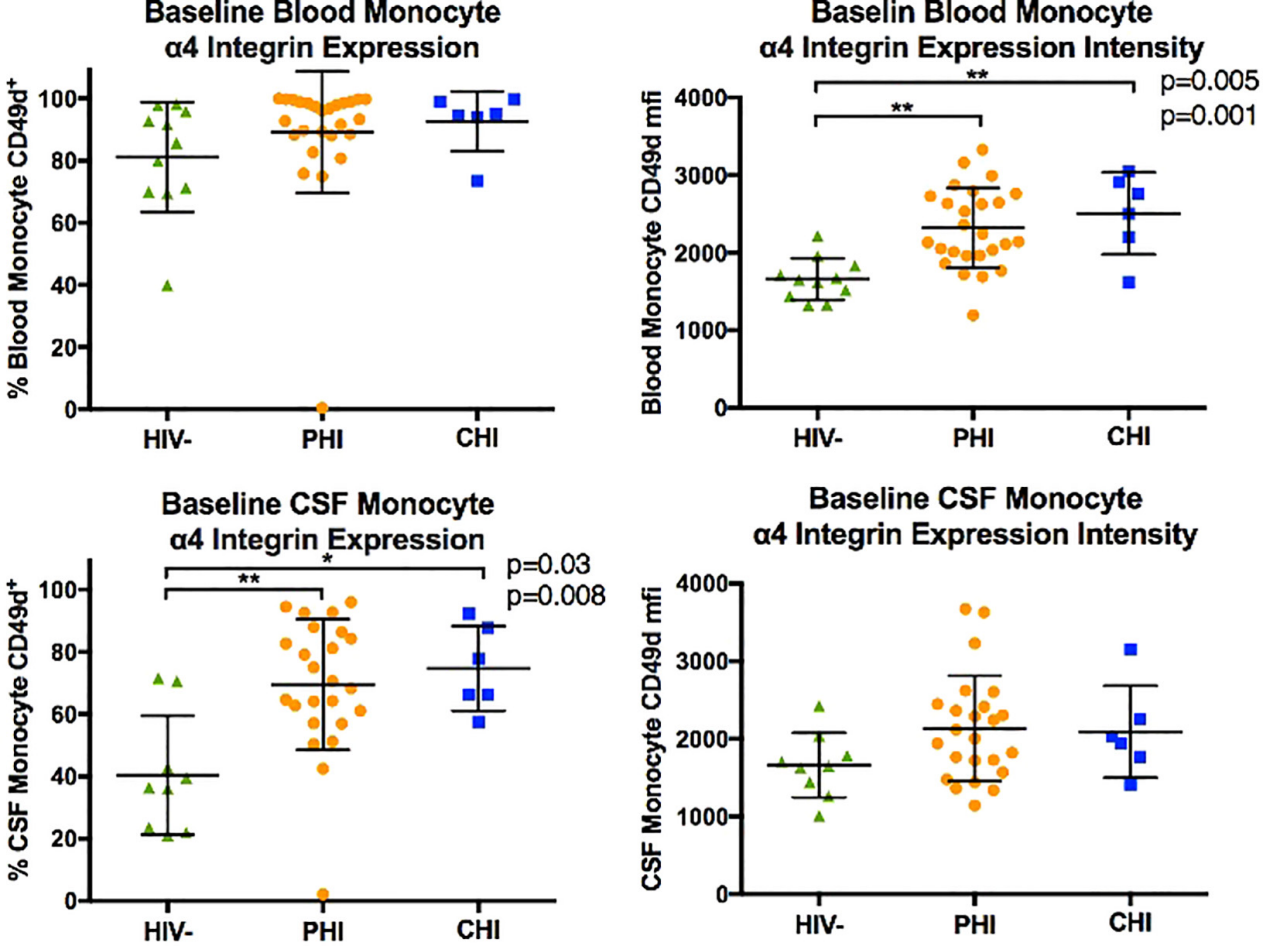
Comparison of monocyte α4 integrin expression (in % CD49d^+^ monocytes and CD49d mean fluorescence intensity, MFI) in blood and CSF of HIV-negative, PHI, and CHI participants at baseline. * p< 0.05, ** p< 0.01.

**Figure 2** displays the monocyte subpopulations in blood and CSF samples across the three participant groups, revealing no significant differences in the percentages of non-classical (CD14dim CD16+), intermediate (CD14+ CD16+), and classical (CD14+ CD16-) monocytes in either blood and CSF between groups. However, α4 integrin expression in monocytes, as determined by the percentage of monocytes expressing α4 integrin and MFI-based α4 integrin expression intensity differed by HIV status (**Figure 3**). Specifically, compared to PWoH, PHI and CHI participants exhibited higher α4 integrin MFI in blood monocytes (p=0.001 and p=0.005) and a higher percentage of α4 integrin expression in CSF monocytes (p=0.008 and p=0.03).

### Longitudinal immunological changes in blood and CSF samples in untreated PHI participants

#### CD4+ and CD8+ T-cell activation

**Figure 4:**
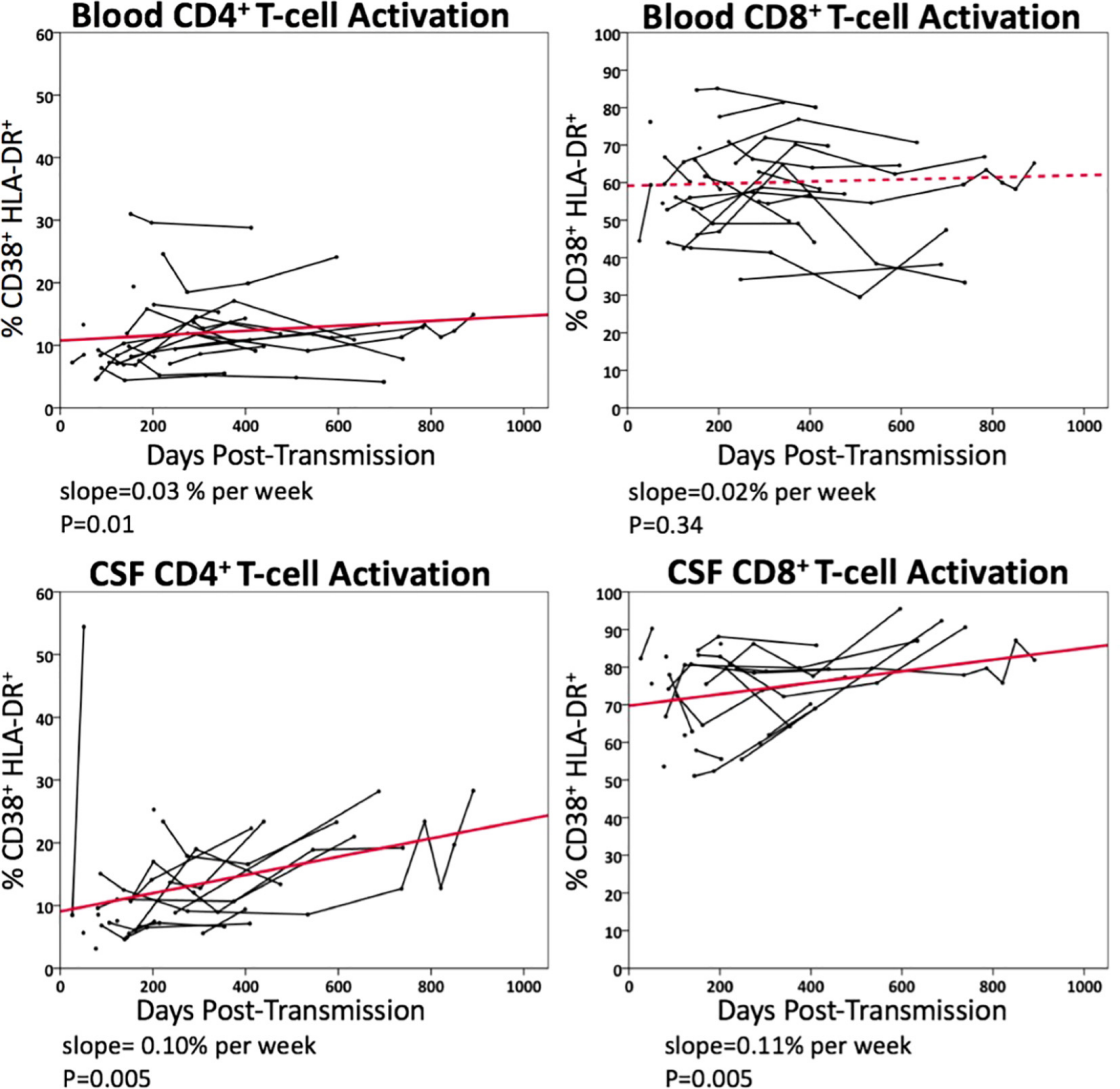
Longitudinal changes in blood and CSF CD4^+^ and CD8^+^ T-cell activation (% CD38^+^HLA-DR^+^) in untreated PHI participants (n=21). Data points from the same participant over time are connected. Solid line represents significant regression slope and dashed line non-significant regression slope.

**Figure 4** illustrates longitudinal percentage changes in activated CD4+ and CD8+ T-cells in blood and CSF samples, based on 113 follow-up samples collected from 21 PHI participants who had not initiated ART during the period. In the blood, activated CD4+ T-cells increased by 0.03% per week (p=0.01), while activated CD8+ T-cells did not show a significant increase (p=0.34). In CSF, activated CD4+ T-cells increased by 0.10% per week longitudinally (p=0.005), while activated CD8+ T-cells increased by 0.11% per week (p=0.005). These findings highlight a greater increase in activated CD4+ and CD8+ T-cells in the CSF compared to plasma during this period. Additionally, activated T-cells were more frequently observed in the CSF than in plasma, up to a three-fold difference.

### Monocyte subpopulation and α4 integrin expression

Compared to the significant increases in the percentages of activated CD4+ and CD8+ T-cells in blood and CSF samples, mixed model analysis did not reveal significant changes in the composition of intermediate, classical, and non-classical monocyte subpopulations in either sample over time. Furthermore, metrics of α4 integrin expression in blood and CSF monocytes, whether by expression percentage or by MFI, did not statistically differ over time.

### Longitudinal immunological changes in blood and CSF samples following ART initiation

#### CD4+ and CD8+ T-cell activation

**Figure 5:**
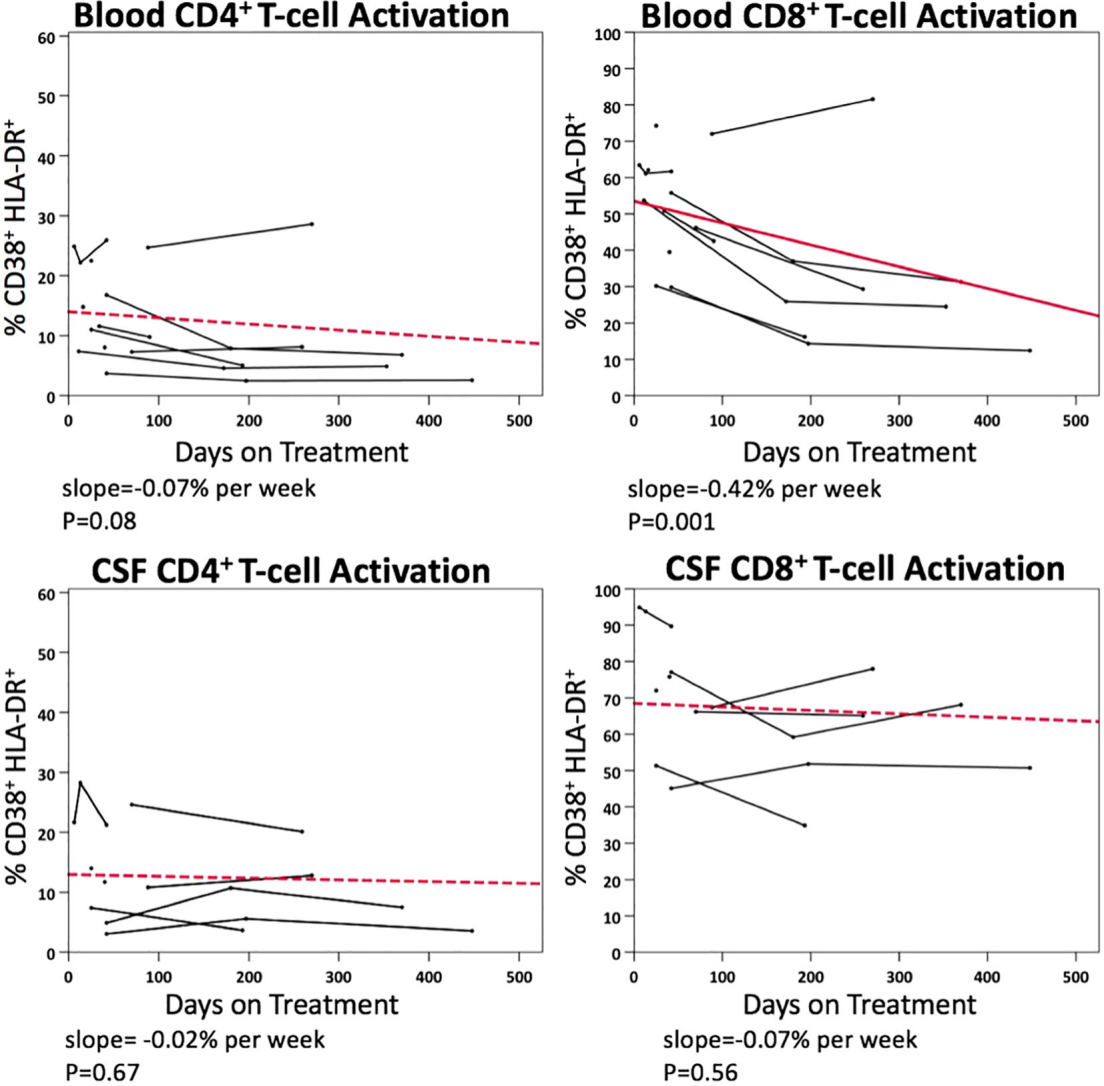
Longitudinal changes in blood and CSF CD4+ and CD8+ T-cell activation (% CD38^+^ HLA-DR^+^) in PHI participants (n=11) after ART.

**Figure 5** illustrates the longitudinal changes in CD4+ and CD8+ T-cell activation in blood and CSF samples following ART initiation, based on 55 paired samples from 11 PHI participants. Following ART initiation, activated CD4+ T-cell level did not significantly decline in blood (p=0.08), while activated CD8+ T-cell level declined at a rate of 0.42% per week (p<0.001). No statistically significant decline in activated CD4+ or CD8+ T-cells was observed in CSF samples after ART.

### Monocyte subpopulation and α4 integrin expression

Following ART initiation, neither blood nor CSF samples exhibited a significant change in the composition of monocyte subpopulations. Additionally, α4 integrin expression in monocytes in blood and CSF remained generally unchanged, except a reduction in MFI-based α4 integrin expression intensity in blood monocytes over time (p=0.03).

### Correlations between HIV RNA, T-cell activation and monocyte subpopulations in CSF

Potential association between CSF HIV RNA, CD4+ and CD8+ T-cell activation and metrics of monocytes in the CSF samples were explored using a mixed model longitudinal data analysis (**Table 2**). Before ART initiation, percentages of activated CD4^+^ T-cells (p<0.001) and activated CD8^+^ T-cell (p=0.013) in CSF correlated with CSF HIV RNA levels in univariate analyses. In multivariate analyses, CSF CD4+ T-cell activation (p=0.005), but not CD8+ T-cell activation (p=0.45), remained independently associated with CSF HIV RNA, demonstrating significant mediation effects that explained 69% of the association in the predictive model (**Supplementary Figure S1**). In contrast, neither the percentages of monocyte subpopulations nor the metrics of α4 integrin expression in CSF correlated with CSF HIV RNA pre-ART in the univariate analysis (not shown).

**Table 2:** CSF CD4+ and CD8+ T-cell activation (% CD38^+^ HLA-DR^+^) as univariate and multivariate predictors of CSF viral load (log_10_ CSF HIV RNA).

Univariate Analysis			
Variable 1	Variable 2	Regression Coefficient	p-value
CSF CD4^+^ T-cell activation	CSF HIV RNA (log_10_ copies/mL)	0.0436	0.0003
CSF CD8^+^ T-cell activation	CSF HIV RNA (log_10_ copies/mL)	0.0274	0.013
CSF CD8^+^ T-cell activation	CSF CD4^+^ T-cell activation	0.496	<0.0001

Multivariate Analysis			
Variable 1	Variable 2	Regression Coefficient	p-value
CSF CD4^+^ T-cell activation	CSF HIV RNA (log_10_ copies/mL)	0.0383	0.005
CSF CD8^+^ T-cell activation		0.0088	0.45

*T-cell activation = % CD38^+^ HLA-DR^+^

### Correlations between T-cell activation and measures of monocytes before and after ART initiation

Potential correlations between CD4+ and CD8+ T-cell activation and metrics of monocytes in the blood and CSF samples were explored in pre-ART and post-ART settings. Prior to ART initiation, correlations between monocyte metrics and T-cell activation were generally absent in both blood and CSF samples, except positive but modest correlations between monocyte α4 integrin MFI and the frequencies of activated CD4+ T-cells (b=0.004, p=0.02) and activated CD8+ T-cells (b=0.008, p=0.01) in the blood (**Supplementary Table S3**).

In blood, following ART initiation, the frequency of intermediate monocytes increased with the percentages of activated CD4+ T-cells (b=0.22, p=0.02) and CD8+ T-cells (b=1.4, p<0.001), while the frequency of classical monocytes was negatively associated with the percentages of CD4+ T-cells (b=-0.19, p=0.03) and CD8+ T-cells (b=-1.14, p=0.001) (**Supplementary Table S4**). Additionally, the frequency of α4 integrin expression and α4 integrin MFI in monocytes exhibited opposite directions of correlation with CD4+ and CD8+ T-cells activation in blood. The former was negatively associated with the frequencies of activated CD4+ T-cells (b=-0.21, p=0.007) and CD8 T-cells (b=-0.69, p=0.02), while the latter was positively associated with the frequencies of activated CD4+ T-cells (b=0.005, p=0.01) and CD8+ T-cells (b=0.018, p=0.01). In CSF, none of the monocyte parameters were associated with T-cell activation, except a positive and modest correlation between α4 integrin MFI and frequencies of activated CD4+ T-cells (b=0.014, p=0.005).

## Discussion

Leveraging a longitudinal collection of paired blood and CSF samples since PHI diagnosis, this study aimed to investigate the evolution of immune cell activation in blood and CSF compartments during early HIV infection, before and after ART initiation. Previous studies suggest that immune activation in the CSF compartment occurs following HIV CNS invasion, defined as detection of HIV RNA in CSF. In one study, CSF pleocytosis occurred following the detection of HIV RNA in CSF (33). Another study reported a significant increase in activated CD8+ T-cells in CSF during Fiebig stage III of AHI, though no statistical difference was found between the percentages of activated CD8+ T-cells in CSF from Fiebig stages I-II of AHI and PWoH (14). Importantly, while there was a notable rise in the percentage of activated CD8+ T-cells in CSF during Fiebig stage III of AHI, the level of activated CD8+ T-cells remained lower than those seen in PWH with untreated CHI (14).

### T-cell activation in PHI before and after antiretroviral therapy during PHI

In the current study, measurements of activated CD4+ and CD8+ T-cells in the paired samples revealed similar frequencies of activated CD4+ and CD8+ T-cells in blood between untreated PHI and CHI participants. However, levels of CD4+ and CD8+ T-cell activation in CSF during PHI were intermediate between those of CHI and PWoH, without reaching statistical significance compared to either group. During longitudinal follow-up prior to initiation of ART, PHI participants exhibited progressive increases in activated CD4+ and CD8+ T-cells in the CSF, but not in the blood. These findings align with published studies indicating that T-cell activation in the CSF is relatively delayed compared to activation in the systemic circulation, and data from this cohort that soluble immune activation markers continue to increase over time during early HIV untreated infection (20).

In this study, following ART initiation, levels of T-cell activation declined in both blood and CSF compartments. However, only the decline of activated CD8+ T-cell in blood reached statistical significance, whereas the corresponding decline in CSF was relatively modest (0.42% vs 0.07% per week). While the lack of significant change could be related to small sample size, the findings may indicate that T-cell activation in the CSF, and potentially intracerebral inflammation, requires a prolonged duration to normalize if it ever reaches levels comparable to those in PWoH. In ART-naïve PWH with CHI, especially in those with advanced immunodeficiency and HIV encephalitis, a protracted course of decay in HIV RNA in the CSF compared to blood is frequently observed (34), potentially contributing to a slower course of decline in activated CD8+ T-cells in the CSF. However, this phenomenon would not be applicable to the current study, as all PHI participants commenced ART before the development of advanced immunodeficiency or overt neurological manifestations.

### Monocyte subpopulation and α4 integrin expression in PHI before and after antiretroviral therapy

Monocytes may be susceptible to HIV infection and contribute to long-term viral reservoirs as tissue macrophages (35). Monocytes can be classified into three subtypes: CD14^dim^CD16^+^ non-classical monocytes, CD14^+^CD16^+^ intermediate monocytes, and CD14^+^ CD16^-^ classical monocytes. In PWH, various monocytes measures have been associated with adverse CNS outcomes. For instance, higher levels of circulating intermediate monocytes and intact HIV reservoir in monocytes in blood were associated with worse cognitive function in virally-suppressed women with HIV (36, 37), whereas non-classical monocyte levels in blood were negatively correlated with cerebral small vessel disease and cognitive performance (38). Additionally, monocyte activation markers, such as sCD14 and sCD163, are frequently elevated in PWH with cognitive impairment compared to those without (39).

Prior studies of AHI and PHI have highlighted elevations of monocyte activation markers in blood and CSF shortly after HIV acquisition (3, 4). However, there has been less investigation into changes in monocyte subpopulations and their expression of trafficking markers in both compartments. α4 integrin, also known as very late antigen 4 (VLA-4) or CD49d, is a subunit of the transmembrane integrin protein that mediates immune cell adhesion to endothelial cells, facilitating their extravasation from the blood into other tissue compartments including the CNS (40, 41). In a prior study, application of anti-α4 antibody that blocks monocyte/macrophage trafficking to the brain and gut in late Simian immunodeficiency virus (SIV) infection reduced and stabilized neuronal injury in non-human primate model (42). However, the potential benefit of alpha-4 blocker on direct virologic control in the SIV model remains unclear (43, 44).

In the current study, the composition of monocyte subpopulations did not differ by HIV status in either compartment when assessed cross-sectionally. Additionally, no significant changes in the composition of monocyte subpopulations were observed in blood and CSF samples during the longitudinal follow-up of pre-ART PHI participants. The findings contrast with a previous study that investigated the composition of blood monocyte subpopulations in PWH. That study reported elevated levels of intermediate monocytes and reduced levels of classical monocytes in blood samples from pre-ART PWH with Fiebig stages III-V AHI and CHI (45). The findings from that study suggest that similar changes are likely to occur during PHI, as it is chronologically positioned between AHI and CHI. To date, the understanding on the impacts of HIV on compartmental monocyte subpopulations remains limited, with challenges including the confounding effects from co-infections and chronic conditions. For instance, syphilis infection (46) and the use of methamphetamine (47), which are not uncommon in PWH, can individually affect the composition of monocyte subpopulations in the blood. Another study reported differing composition of monocyte subpopulations in blood and CSF between HIV-subtypes (48).

Compared to monocyte subpopulation composition, the expression of α4 integrin in monocytes differed between PWH and PWoH. Pre-ART PHI and CHI participants showed higher levels of α4 integrin MFI in blood monocytes and a greater percentage of α4 integrin expression in CSF monocytes than PWoH. Moreover, this elevated α4 integrin expression in blood and CSF monocytes persisted over time in PHI participants, though without significant progression. Our exploratory analysis further suggests that α4 integrin expression in monocytes is associated with T-cell activation, particularly in the blood compartment and after ART initiation. Future studies should investigate the role of α4 integrin expression in monocytes in residual immune activation in PWH on stable ART.

This study has limitations. The longitudinal analysis was based on a relatively small sample size of paired blood and CSF samples from people with PHI, with variation in the timing of ART initiation. In addition, longitudinal sampling of participants with CHI was not included, limiting our ability to compare the impact of early versus late ART initiation on T-cell activation and monocyte subpopulations. The duration of HIV infection was estimated through combined sequential HIV-related serological changes and clinical history acquisition, however accurate estimation is often challenging and exact durations are not available. The impacts of other potential modifiers on study outcomes in the participants who started ART, such as CNS-penetrating efficacy of ART and the degree of immune recovery (e.g., individual improvement in CD4+ T-cell counts), were not determined because of the small sample size in this category (n=11). Finally, this analysis did not assess associations between cellular immune measures and clinical outcomes such as neuropsychological performance or daily function.

## Conclusions

This study of paired CSF and blood samples from young male participants initially studied in the first year of HIV acquisition and followed longitudinally pre and post-ART demonstrates a compartmentalized immune response in the CNS as compared to blood throughout early infection and treatment. While we observed a delay in emergence of T-cell activation in the CNS compartment compared to during untreated PHI, overall we found a higher frequency of CD8+ T cell activation in the CSF, and increasing frequencies of CD4+ and CD8+ T cell activation in the CSF during early infection prior to ART. This did not contemporaneously follow responses in blood, where CD4+ T cell activation but not CD8+ T cell activation mildly increased during this period. Our study also observed a slower decline of CD8+ T-cell activation in the CSF compared to blood after ART initiation, indicating the presence of persistent intrathecal immune activation despite ART initiation during early stages of HIV infection. Finally, frequency of CSF CD4+ T-cell activation but not CD8+ T-cell activation or monocyte sub-phenotypes was independently associated with CSF HIV RNA prior to ART, suggesting that in the early stages of infection, T cell infection is an important determinant of viral replication within the CNS. Compartmentalized cellular CNS immune activation occurs and progresses during early HIV infection and is not immediately ameliorated by ART.

## Data Availability

The raw data supporting the conclusions of this article will be made available by the authors, without undue reservation.
