# Supplementary material for: Longitudinal CNS and systemic T-lymphocyte and monocyte activation before and after antiretroviral therapy beginning in primary HIV infection
Source: Front Immunol. 2025 Feb 25;16:1531828. doi: 10.3389/fimmu.2025.1531828 (PMC11893981; doi:10.3389/fimmu.2025.1531828)
Supplement: Supplementary file 1 [file DataSheet1.docx]

**SUPPLEMENTARY TABLES AND FIGURES**


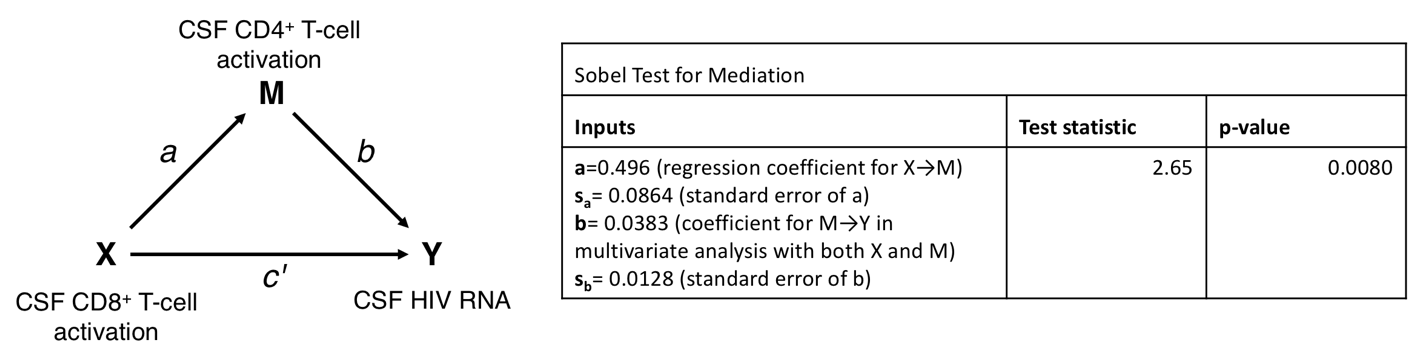


**Figure S1.** Sobel test for mediation of the association between CSF CD8^+^ T-cell activation (independent variable) and CSF HIV RNA levels (dependent variable) by CSF CD4^+^ T-cell activation (mediator).
