# Supplementary material for: Longitudinal CNS and systemic T-lymphocyte and monocyte activation before and after antiretroviral therapy beginning in primary HIV infection
Source: Front Immunol. 2025 Feb 25;16:1531828. doi: 10.3389/fimmu.2025.1531828 (PMC11893981; doi:10.3389/fimmu.2025.1531828)
Supplement: Supplementary file 2 [file Table1.docx]

**Table S1.** Flow cytometry markers for T-cell and monocyte panels 1 and 2

|  | Panel 1 | Panel 2 |
| --- | --- | --- |
| T-cell | CD3  CD4  CD8  CD38  HLA-DR  CCR5  CXCR3 | CD3  CD4  CD8  CD38  HLA-DR  CD27  CD28  RA |
| Monocyte | CD3  CD4  CD14  CD16 | CD45  CD14  CD16  CD49d |
