# Supplementary material for: Longitudinal CNS and systemic T-lymphocyte and monocyte activation before and after antiretroviral therapy beginning in primary HIV infection
Source: Front Immunol. 2025 Feb 25;16:1531828. doi: 10.3389/fimmu.2025.1531828 (PMC11893981; doi:10.3389/fimmu.2025.1531828)
Supplement: Supplementary file 3 [file Table2.docx]

**Table S2.** Participant demographics and visit information for flow cytometry panels 1 and 2

|  | Total | Panel 1 | Panel 2 |
| --- | --- | --- | --- |
| Number of participants (n) | 51 | 21# | 38# |
| Enrollment years | 2005-2010 | 2005-2008 | 2008-2010 |
| Total visits | 168 | 89 | 79 |
| Pre-ART | 113 | 66 | 47 |
| Post-ART | 55 | 23 | 32 |
| Estimated time post HIV transmission (months) | 3.4 (1.9, 5.6) | 4 (2.7, 5.3) | 2.8 (1.8, 5.8) |
| Follow-up duration | 6.9 (1, 22.9) | 7.4 (2.1, 20) | 6.4 (0, 24) |
| #A small subset of PHI participants had samples run on both panel 1 & 2. | | | |
