# Supplementary material for: Longitudinal CNS and systemic T-lymphocyte and monocyte activation before and after antiretroviral therapy beginning in primary HIV infection
Source: Front Immunol. 2025 Feb 25;16:1531828. doi: 10.3389/fimmu.2025.1531828 (PMC11893981; doi:10.3389/fimmu.2025.1531828)
Supplement: Supplementary file 4 [file Table3.docx]

**Supplementary Table S3.** Correlations between metrics of monocytes and other outcomes variables in PHI participants pre-ART
