# Supplementary material for: Longitudinal CNS and systemic T-lymphocyte and monocyte activation before and after antiretroviral therapy beginning in primary HIV infection
Source: Front Immunol. 2025 Feb 25;16:1531828. doi: 10.3389/fimmu.2025.1531828 (PMC11893981; doi:10.3389/fimmu.2025.1531828)

**Supplementary Table 4.** Correlations between metrics of monocytes and other outcomes variables in PHI participants after ART.


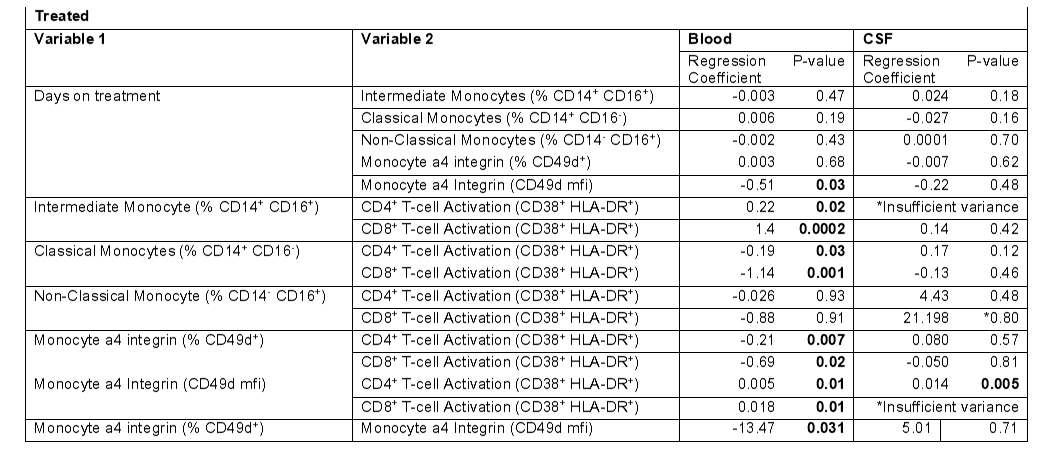

Supplement: Supplementary file 5 [file Table4.docx]
